# Development of the muographic tephra deposit monitoring system

**DOI:** 10.1038/s41598-020-71902-1

**Published:** 2020-09-09

**Authors:** Hiroyuki K. M. Tanaka

**Affiliations:** Earthquake Research Institute, The University of Tokyo, 1-1-1 Yayoi, Bunkyo, Tokyo, 113-0032 Japan

**Keywords:** Solid Earth sciences, Volcanology, Physics, Particle physics

## Abstract

Measurements of volcanic tephra fallout deposits provide useful information about the magnitude and intensity of explosive volcanic eruptions and potential for remobilization of deposits as dangerous volcanic flows. However, gathering information in the vicinity of erupting craters is extremely dangerous, and moreover, it is often quite difficult to determine deposit thickness proximal to volcanic craters because the thickness of the deposit is too great to easily measure; thus, airborne remote sensing technologies have generally been utilized during the intermission between eruptions. As an alternative tool, a muographic tephra deposit monitoring system was developed in this work. Here we report the performance of this system by applying the muographic data acquired at Sakurajima volcano, Japan as an example. By assuming the average density of the deposit was 2.0 g cm^−3^, the deposit thicknesses measured with muography were in agreement with the airborne results, indicating that volcanic fallout built up within the upper river basin, showed its potential for monitoring the episodic tephra fallouts even during eruptions.

## Introduction

Explosive volcanic eruptions produce tephra fallout, consisting of juvenile magma and older rock fragments that bring multi-aspect effects to the atmosphere and land surface^1^. The unconsolidated tephra fallout deposits in the river basin can evolve to form rainfall-triggered lahars (RTLs), which pose a significant hazard to downstream infrastructure^2^. Lahars are gravity-driven, rapid movements of material composed of a mixture of rock debris and water (in flux > 10^3^ m^3^ s^−1^), which pose significant secondary hydrologic hazards^3^. RTLs can often bury floodplains and devastate the environment located at downstream areas of rivers^4^. After the continuous discharge of volcanic ash and gas for extended periods, the surrounding vegetation is lost, and the land surface is covered with fine-grained volcanic ash. The vegetation losses reduce soil stability^5^, and ash deposits reduce the surface permeability. As a consequence, the erosion of the land surface is accelerated by the surface runoff, and rills and gullies are developed^6^ in particular on steeper slopes^7^. This geographical alteration allows lahars to be triggered by smaller rainfalls. Relationships between post-eruption RTLs and rainfall intensity/duration have been studied at Colima, Mexico^8^, Melapi, Indonesia^9^ and Pinatubo, Philippines^10^. They all show a power-law relationship between intensity and duration of rainfalls that triggered lahars. The RTL initiations are controlled by many variable factors including (A) deposit thickness^11^, (B) compaction^11^, (C) soil grain size distribution^12^, (D) vegetation cover^5^, (E) rainfall intensity and duration^13^, (F) antecedent rainfall^14^, and (G) the slope angle of the land^12^. There are other motivations to know the thicknesses of tephra deposits and their granulometry^15,16^. For example, volcano scientists try to determine the magnitude of eruptions by integrating thickness measurements^17^. Muography gives a continuous record of thickness variation; hence a potential to improve these estimates. Near volcanic vents, thickness is usually constrained to a few measurements using traditional methods^18^. Ground penetrating radar is sometimes used to determine deposit thickness, but cannot be done remotely^18^.

Airborne laser scanning measurements have been conducted to provide alternative information on the deposit and erosion at erupting volcanoes^19^. However, due to the high costs, frequent airborne measurements are impractical.

Developments of tephra deposits can be visualized with muography by taking advantage of the strong penetration power of cosmic-ray muons. The principle of muography is similar to medical applications of radiography except muons are utilized instead of x-rays. Both muography and radiography visualize the shadow that is created when a part of the irradiated probes (muons or x-rays) are stopped by dense materials located inside the target objects. However, unlike X-rays, muons are a natural resource that can be universally utilized everywhere on Earth because they are generated in the atmosphere via the decay of mesons which are generated by the collision between primary cosmic rays and atmospheric nuclei^20^. The resultant muons arrive only from the upper hemisphere, and their vertical flux is 70 m^−2^ s^−1^. While vertical muons can be utilized for imaging a target object located right above the detector, topographically prominent objects such as volcanoes are also targetable by detecting muons at angles that are nearly horizontal. Galactic cosmic rays (GCRs), parent particles of muons, are accelerated by energetic events in the galaxy and are deflected multiple times by the random components of the galactic magnetic field before arriving at the Earth. By the time GSRs arrive at the Earth, the initial directional information has been completely lost; resultant muons with sufficiently high energy are azimuthally isotropic. However, the muon energy spectrum varies for different arriving zenith angles because the generated meson’s mean free path differs in the atmosphere. The average energy of vertical muons is lower because the parent mesons collide with atmospheric nuclei before they decay while that of horizontal muons is higher because the parent mesons decay before they collide with atmospheric nuclei. As a result, horizontal muon flux is lower than vertical flux, but the average muon energy is higher. This high-energy characteristic of horizontal muons enables us to image volcanoes within reasonable timeframes that range from a week to a few months, depending on the size of the volcano. By combining the muon energy spectrum^20^ with the muon energy-range relationship^21^, the transmitted muon flux can be derived. If the average density (*ρ*) is given, the exterior shape of the target volume will determine the densimetric rock thickness along the muon path (*X*) that can be defined by *ρx*, where *x* is the geometrical rock thickness along the muon path. Once *X* is known, the minimum muon energy (*E*_c_) can be derived by using the range-energy relationship. Therefore, the transmitted (survival) muon flux can be derived by integrating the muon energy spectrum with the energy over the range between *E*_c_ and infinity. Inversely, the average density along the muon path can be derived from the transmitted muon flux. Muography was first applied by George^22^ to investigate rock density above a tunnel. Alvarez et al.^23^ tried to image the internal structure of an Egyptian pyramid with spark chambers. Muography is now extensively applied to volcano studies in Japan^24–31^, Italy^32–34^ and France^35,36^. While most muographic images depicting static structures have been generated with data that had been collected in the time scale of many months, there are a few experiments with much shorter time scales enabling time sequential results that indicate the processes within, for example: the explosion of a part of the solidified magma deposited on the crater floor^37^, ascent and descent of magma underneath the crater floor^38^, and enlargement of the vent size and crack generation by recurrent fumarolic explosions^39^. The muography-based remote monitoring system that was developed in this work will expand the applicability of muography to the volcanology field as an additional monitoring tool. In this work, we developed a muography-based remote monitoring system to estimate the location and thickness of tephra deposits. The performance of this system was evaluated by inputting the data collected between 2014 and 2017 at Sakurajima as an example.

## Results

### Data

**Figure 1:**
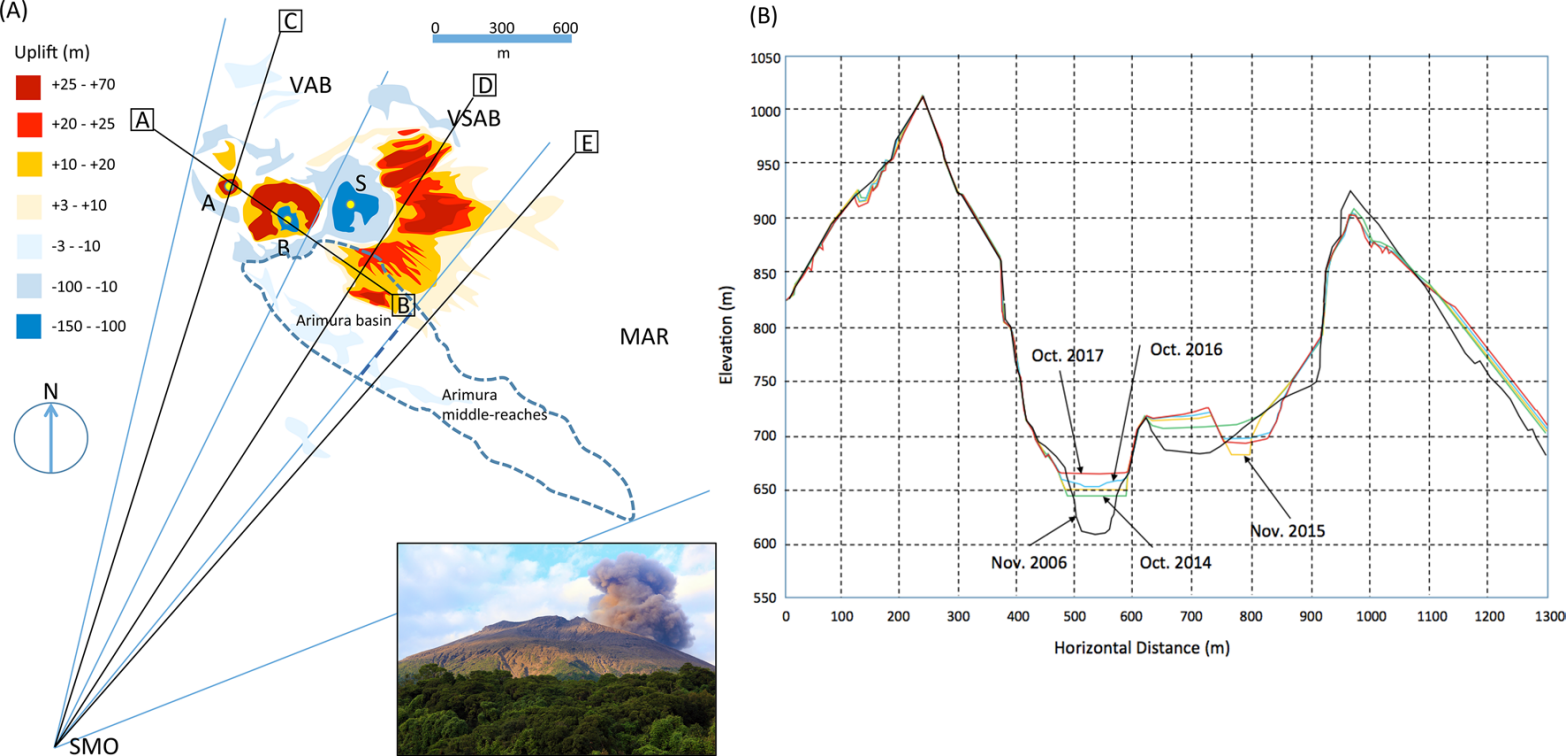
Geometrical configuration of the current observation. (**A**) The locations of the observation station and vents are shown. A, B, S, and SMO respectively indicate Vent A, Vent B, Showa Vent, and the Sakurajima Muography Observatory. Yellow circles indicate the location of these vents. VAB, VSAB, and MAR indicate the angular regions (Vent A and Vent B, Showa Vent and Arimura Basin, and the middle reaches of Arimura River) that correspond to Fig. 5. The color-plots show the deposition and erosion of the ground as measured between November, 2006 and November, 2017. The region surrounded by dashed lines was defined as Arimura Basin by the Japan Meteorological Agency (JMA)^19^, but in this work, this region was divided into two regions that were respectively defined as Arimura Basin and the Arimura middle-reaches. Cross-sectional views along SMO-C, SMO-D, and SMO-E lines are shown in Fig. 2. The inset shows a photograph of the volcano taken from the SMO. (B) The time-dependent cross-sectional view of Central Crater (including Vent A and Vent B) of Sakurajima volcano along the A-B line in (A). All of the plots were produced by the author based on the JMA report^19^. The author (H.K.M.T) drew these maps with Microsoft PowerPoint, Slide Presentation Software, and holds the copyright.

Sakurajima, located in Kyushu Island, Japan, is one of the most active volcanoes in the world and has been continuously active since 1955. Most local vegetation has been completely destroyed by volcanic gas and ash flows. RTLs have frequently occurred and caused debris flows in the downstream alluvial fan. There are three vents at the top of Minami-dake, the main scoria cone of Sakurajima volcano: Vent A, Vent B, and Showa Vent. Vent A and Vent B are located in Central Crater, and Showa Vent is located in an adjacent smaller crater. The volcano has erupted about 8,000 times between 2009 and 2017 (~ 3 times/day on average) either from Vent B or Showa Vent. During eruptions, large volumes of volcanic ejecta have been released, and the episodic nature of eruptions have caused rapid sediment uplift around the vent areas, generating 5.2 × 10^6^ m^3^ of sedimentation in total in the vicinity of Vent B and Showa Vent after repeated deposition and erosion processes (7.2 × 10^6^ m^3^ of deposition and 2.0 × 10^6^ m^3^ of erosion) during the last 11 years^19^ (Fig. 1). At Sakurajima, the airborne laser scanning measurements have been conducted every year to capture the three-dimensional topography of the crater and other steep regions (Fig. 1). The intermissions of eruptions have been carefully chosen and a manned helicopter was operated above the active crater regions. An enlargement of the crater caused significant subsidence near Showa Vent during the beginning of the eruption episode, and after October 2016, variations in the depth and diameter of Showa Vent have been small; the maximum subsidence measuring 5 m was observed at the western rim of the crater. The airborne results were employed to estimate the total mass deposit accumulated between November 2014 and October 2016 within the angular regions of Vent A, Vent B, Showa Vent, and Arimura Basin. The height variations (Fig. 1B) and the area colored in yellow (and blue) (Fig. 1A) were used for calculating the mass of deposition (and erosion) by assuming the uniform density of 2 g cm^−3^. The detailed results will be discussed in Discussion section.

During this data gathering period between December 1, 2014 and November 20, 2016, only Showa Vent was very active (1,433 eruptions in this period), and only a small number of eruptions (13 eruptions) were observed at Vent B. Vent A was dormant. The annual number of eruptions from the active vents (Vent A and Vent B) was 656 in 2014, and it increased to 1,252 in 2015, but decreased to 153 in 2016. During the period between February 2015 and May 2015, the eruption frequency was exceptionally higher than other periods (5–9 times/day on average). During this period, the Japan Meteorological Agency (JMA) conducted tephra fallout measurements in Kagoshima city, Japan, and modeled the total amount of the ejected ash to be ~ 3.6 × 10^9^ kg in these four months^40^. Currently, it is legally restricted to enter Sakurajima volcano, including for the purpose of research. The closest location where the tephra fallout measurements could be conducted was Kagoshima-city that was located 10 km from the active vent. 86 lahars observed between 2014 and 2015 were all triggered by rainfalls stronger than 10 mm/h^41^.

There are two major rivers at Sakurajima: Arimura River and Kurokami River. Based on the repeated airborne laser scanning measurements, it was estimated that tephra with volumes of 1.07 × 10^6^ m^3^ and 2.02 × 10^6^ m^3^ have respectively deposited and approximately 50% (respectively 5.44 × 10^5^ m^3^ and 1.16 × 10^6^ m^3^) have been eroded away from the Arimura upper river basin (Arimura Basin) and the Kurokami upper river basin (Kurokami Basin) (as respectively indicated by the areas surrounded by dashed lines (R1 and R2) in Fig. 1) between November 2006 and October 2017^19^. Along the Arimura River, most of the subsidence occurred in the Arimura Basin, and uplift was observed only on the platform near Showa Vent because the deposited materials in the valley could be easily washed away. In this period, the averaged deposition rates per year in R1 and R2 were respectively estimated to be 10^5^ m^3^/year and 2 × 10^5^ m^3^/year.

**Figure 2:**
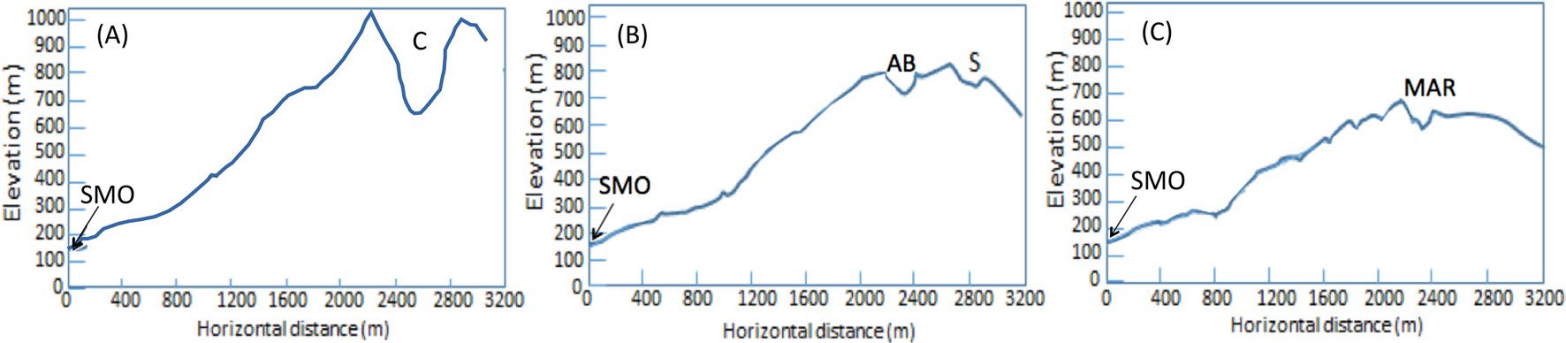
Cross-sectional presentations of the vent region of Minami-dake, Sakurajima. The cross-sectional views are shown along the lines SMO-C (**A**), SMO-D (**B**), SMO-E (**C**) in Fig. 1. Central crater (including Vent A and Vent B), Showa Vent, Arimura basin, Sakurajima Muography Observatory are respectively indicated by C, S, AB, and SMO.

A scintillator-based muography observation system (SMOS) was placed at the Sakurajima Muography Observatory (SMO) (31°N and 136°E at a height of 150 m above sea level). SMO is located at the southern foot of Minami-dake, Sakurajima volcano. At the SMO, a noise-cut uninterruptible power supply (UPS) (Denken-Seiki RI-N) was installed to prevent the interruption of the measurements due to instantaneous power failures or electronics failures by the electric surge. The geometrical configuration for this observation station is shown in Figs. 1 and 2. The valley located south of Showa Vent is Arimura Basin, and the distance between the SMOS and Arimura Basin (AB) is 2.2 km. The distances between the detector and the closest rims of Central Crater (including Vent A and Vent B) and Showa Vent were respectively 2.1 km and 2.7 km. Figure 2 shows cross-sectional representations of Minami-dake for different azimuthal angles. Due to lightning strikes, the SMOS was not operated during the following periods: (1) April 6–8, 2015, April 30-May 7, 2015, (2) December 27, 2015-January 5, 2016, (3) June 19-June 24, 2016, (4) September 20-October 7, 2016. In addition to these five periods, the SMOS shut down 6 times due to instantaneous power failures resulting in 280 min of operation interruption. As a consequence, the duty cycle of the current data taking was 96% during this period.

### Performance of the real-time monitor

**Figure 3:**
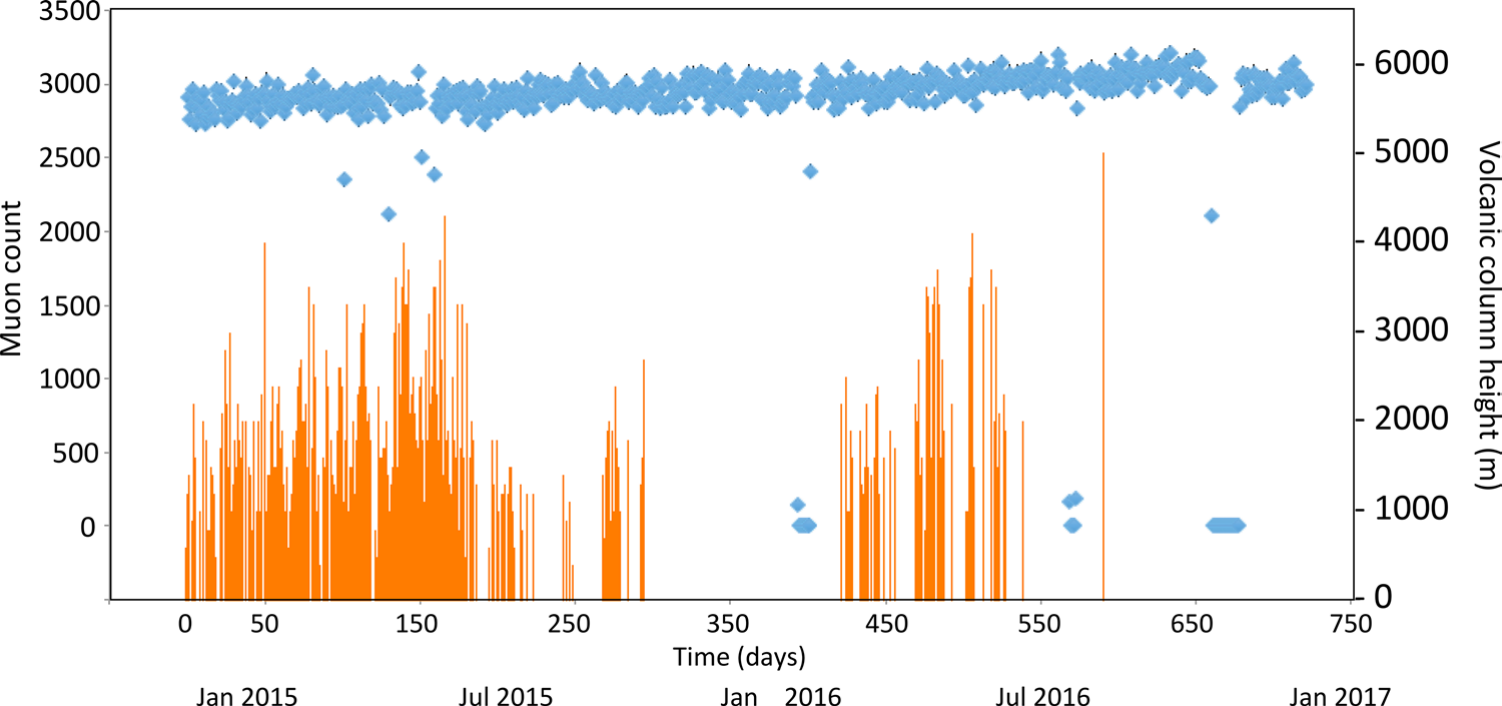
Time sequential plots of the daily open-sky muon-tracking rate (blue rhombus) are shown. The lengths of error bars (1 standard deviation) were smaller than the size of these rhombuses. The heights of volcanic columns (vertical orange bars) are overlaid. Arrows indicate the volcanic explosion events.

The current monitoring system enabled us to remotely monitor the SMOS and confirm its operational status. Figure 3 shows the current monitoring system for the time sequential plot of the daily open-sky muon-tracking rate with the SMOS as observed in the current observation period. The data points displaying zero counts or obviously lower counts than others include the unoperated time periods. The eruption time and the volcanic column height were reported by the Japan Meteorological Agency every day during the current observation period^42^, and their distribution was overlaid to this time-sequential plot so that the comparison between the muon count rate and the volcanic activities was visually possible. The level of the statistics was tolerable for the purpose of the current tephra deposition monitoring. This monitoring system is available online and these aforementioned plots can be easily reproduced by users throughout the world by utilizing the functions already equipped to this system.

### Comparison of the time sequential plots of the muon count rate

**Figure 4:**
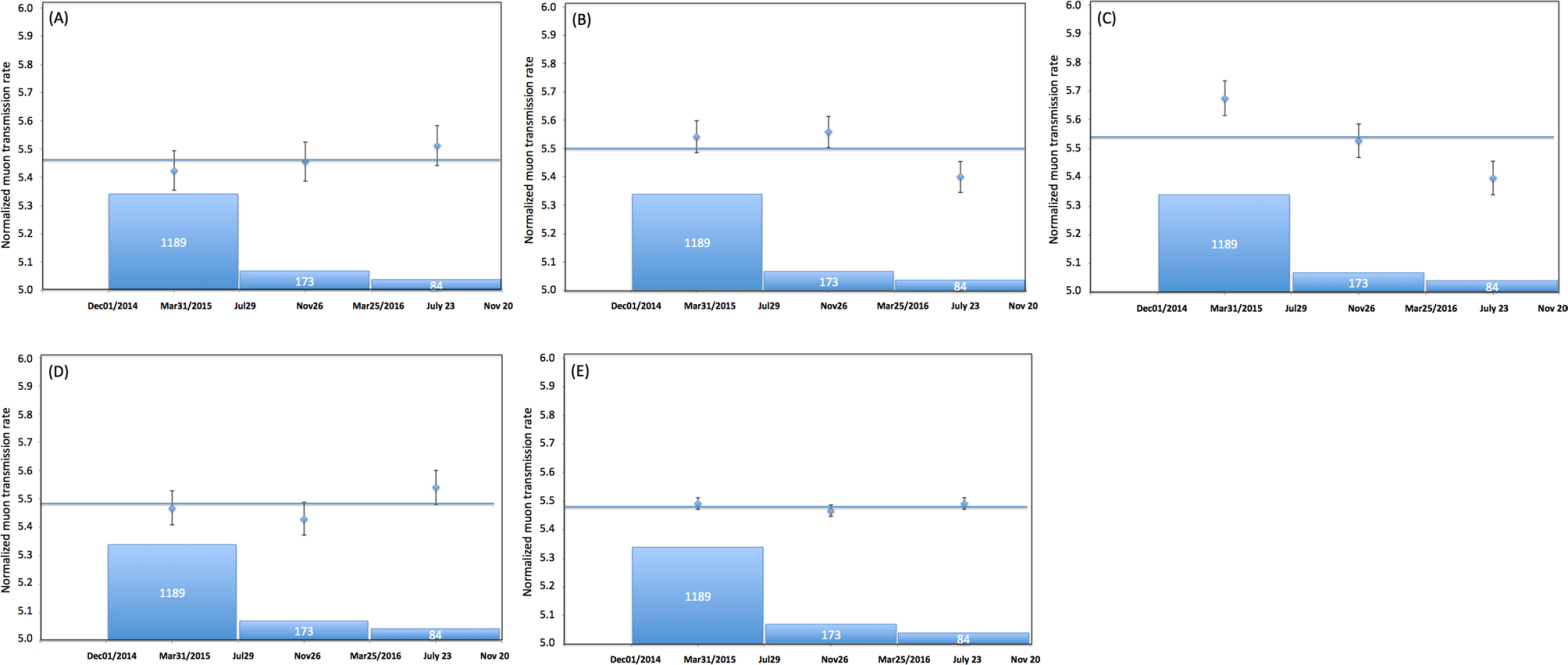
Time sequential variations of the normalized transmitted (survival) muon rate after passing through five angular regions: (**A**) North of Minami-dake (NM), (**B**) Vent A and Vent B (VAB), (**C**) Vent Showa and Arimura Basin (VSAB), (**D**) the middle reaches of Arimura River (MAR), and (**E**) the sky. The bin width is 240 days. The numbers of eruptions corresponding to these bins are also plotted. Vertical bars associated with these data points are error bars (1 standard deviation). Horizontal bars indicate the transmitted muon rate averaged over the current observation period (from December 1, 2014 to November 20, 2016).

**Figure 5:**
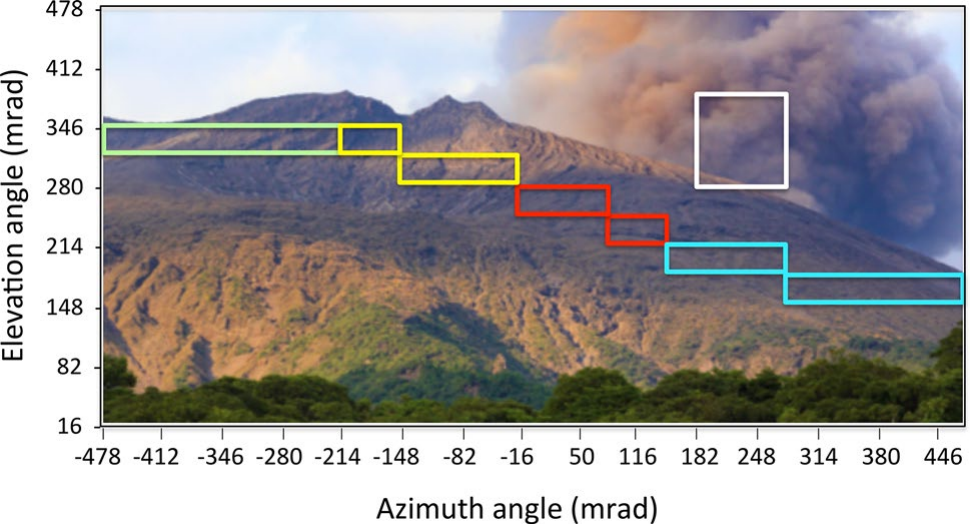
Five angular regions chosen for the current analysis. The green, yellow, red, blue and white boxes respectively indicate the angular regions north of Minami-dake (NM), Vents A and Vent B (VAB), Vent Showa and Arimura Basin (VSAB), the middle reaches of Arimura River (MAR), and the sky. The photograph of Minamidake, Sakurajima Volcano is overlaid.

Figure 4 shows the time sequential variations of the normalized transmitted (survival) muon rate after passing through five angular regions shown in Fig. 5. These regions respectively correspond to north of Minami-dake (NM) (green box), Vent A and Vent B (VAB) (yellow box), Showa Vent and Arimura Basin (VSAB) (red box), the middle reaches of Arimura River (MAR) (blue box), and the sky (white box). The horizontal bin width corresponds to 240 days. The total numbers of events collected during the current observation period were respectively 21,733 (green), 34,475 (yellow), 30,857 (red), 30,397 (blue), 248,706 (white), and 1,540,929 (open-sky). The muon rate in each region was normalized to the open-sky count rate in order to cancel the apparent flux variations due to the data taking failures, and any time-dependent changes in the data collection efficiency. Each of these normalized rates was further divided by each of the total numbers of regionally collected events.

As can be seen in Fig. 4B, one data point collected during the period between July 23, 2016–November 20, 2016 was deviated from the average by 1.81σ. Also in Fig. 4C, the highest (December 1, 2014–January 30, 2015) and lowest (July 23, 2016–November 20, 2016) transmitted muon rates were respectively deviated from the average by 2.34σ and 2.32σ. No statistically significant changes (beyond 1σ) in the transmitted muon rate were observed in other angular regions as shown in Fig. 4A,D,E, and therefore will not be discussed in the following section.

## Discussion

In this work, it was assumed that neither the rock density nor the rock thickness changed during the current observation period after consideration the following factors. (A) Land deformation. In the current observation period (December 1, 2014 to November 20, 2016), the interferometric synthetic-aperture radar analysis was conducted at Sakurajima for the following sets of the dates: (a) September 17, 2014 and April 1, 2015, (b) January 9 and May 15, 2015, (c) January 4 and August 16, 2015, and (d) June 24 and December 09^40,41,43^, however, no subsidence or ascent that exceeded 10 cm was observed throughout the entire volcano including the crater regions in this period. These variations were far below the detection limit of the current observation. (B) Water saturation. The water saturation of the near-surface region due to rainfalls causes high frequency density variations, and thus its soakage and drainage could be averaged over 240 days of the observation period, and as a consequence, this effect was smoothed out. The seasonal variations in the height of the groundwater table were also expected to be mostly averaged over this timescale of the current observation period. These two water effects could not be seen in the near-surface region of the dormant areas (NM and MAR).

The crater floor of Showa Vent ascended by 50 m during the period between October 2014 and November 2015^41^, and it descended by 40 m during the period between November 2015 and October 2016^44^. However, considering the distance from SMO to the closest rim of Showa Vent (2.7 km), these crater floor ascent and descent (196 mrad < *θ* < 214 mrad) were out of the range of VSAB (214 mrad < *θ* < 280 mrad). Additionally, sedimentation deposited beyond Showa Vent was out of the range of the SMOS (see Fig. 2B). On the contrary in the VAB region (Fig. 4B), a statistically significant reduction in the muon count rate was found only in the period between March 25, 2016 and November 20, 2016. This variation pattern was related with the ascent and descent of the crater floor since the distance from SMO to its rim was much closer (2.1 km), and will be discussed further in the next paragraph.

In order to convert the reductions observed in the muon count rate to relative density variations, the following relationship was used. If the variations expected in the densimetric rock thickness are smaller than ± 10%, the resultant variations in the transmitted muon rate can be approximated by the following linear function:

1 $$\Delta X\sim k_{1} \Delta I + k_{2} ,$$

where Δ*I* (m^−2^sr^−1^ s^−1^) is a variation in the penetrating muon flux, *k*_1_ and *k*_2_ are the constants that depend on the muon’s arrival elevation angle (*θ*) and rock thickness (*X* ± Δ*X*) they traverse. The total numbers of events 34,475 and 30,857, respectively having arrived from VAB (1.35 × 10^−2^ m^2^sr) and VSAB (1.125 × 10^−2^ m^2^sr) were recorded in 720 days. Therefore, these muon counts respectively corresponded to the average flux of 4.1 × 10^−2^ m^−2^sr^−1^ s^−1^ and 4.4 × 10^−2^ m^−2^sr^−1^ s^−1^. From these fluxes, the muographically averaged densimetric thicknesses (MADT) were respectively derived to be 890 h g cm^−2^ and 860 h g cm^−2^. The definition of the MADT will be given in Method section. For *θ* = 300 mrad and *X* = 900 h g cm^−2^, the parameters *k*_1_ and *k*_2_ of Eq. (1) were respectively − 1.2598 × 10^−8^ and 1.5385 × 10^−5^.

**Figure 6:**
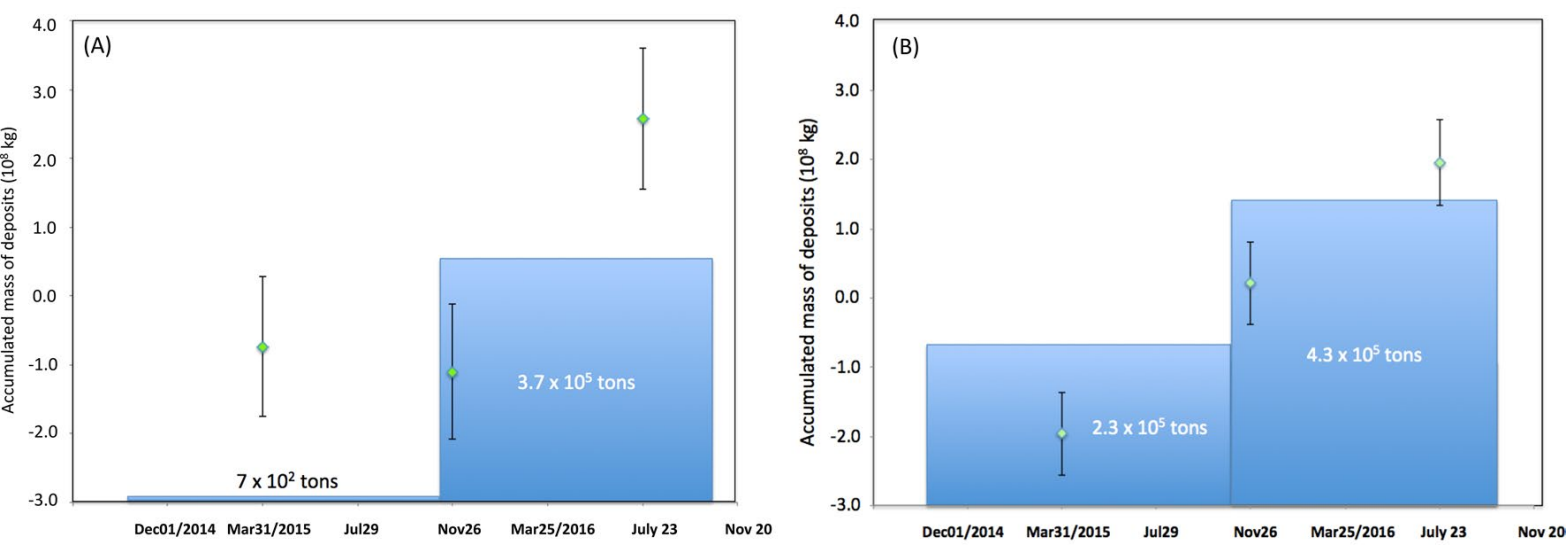
Time sequential variations of the mass of deposits in two angular regions: (**A**) Vent A and Vent B (VAB) and (**B**) Showa Vent and Arimura Basin (VSAB). The bin width is 240 days. The vertical bars associated with these data points are error bars (1 standard deviation). The accumulated mass of the deposits were estimated the airborne thickness measurements and plotted for reference.

**Figure 7:**
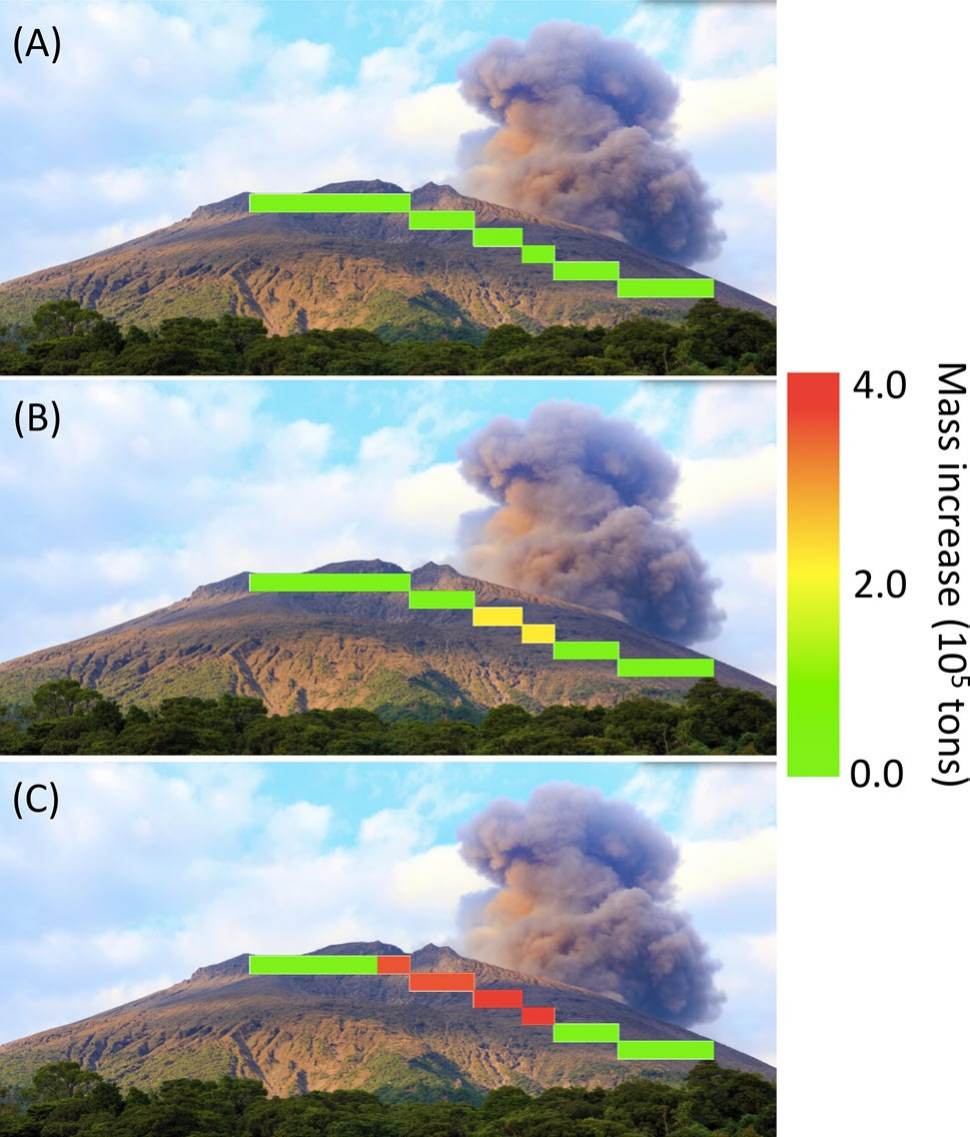
Time sequential mapping of the mass of deposits during the following periods: (**A**) December 1, 2014–July 29, 2015, (**B**) July 30, 2015–March 25, 2016, and (**C**) March 25, 2016–November 20, 2016. The photograph of Minamidake, Sakurajima volcano is overlaid. The volcanic column has risen from Showa Vent in this photograph.

By using Eq. (1), the mass of the tephra (*M*) deposited on VAB and VSAB was calculated as follows:

2 $$M = \left( {\Delta X} \right) \, \times D^{2} \times \Delta \phi \times \Delta \theta \times N,$$

where *D* is the geometrical distance between SMOS and VAB or VSAB, *Δϕ* and *Δθ* are the zenith and azimuth angular bin widths of the muogram, and *N* is the number of bins in each region. The results are shown in Fig. 6. In this plot, the mass derived from the average value in Fig. 4 was set to be zero. The total mass (and mass per unit area) of the deposits in VAB and VSAB since the beginning of this measurement campaign were respectively 3.3 ± 1.4 × 10^8^ kg (1.2 ± 0.5 × 10^4^ kg m^−2^) and 3.9 ± 0.9 × 10^8^ kg (1.8 ± 0.4 × 10^4^ kg m^−2^). In VAB, the rim of Vent B (VBR) was upheaved by 8 m, but the bottom of Vent B subsided by 40 m during the period between October 2014 and November 2015 (Fig. 1B). From these topographic changes, the estimated total mass increased in VAB during this period was 7.0 × 10^5^ kg (considering the upheaved (26,724 m^2^), subsided (5,336 m^2^) areas, and the density assumed to be 2.0 g cm^−3^) that was far below the resolving power of the current monitoring system. However, during the period between November 2015 and October 2016, all of the regions of VAB were uplifted. Vent A, VBR, and Vent B were respectively uplifted by 6 m, 3 m, and 20 m. As a consequence, the estimated total mass (and mass per unit area) increased in VAB in this period was 3.7 × 10^8^ kg (1.6 × 10^4^ kg m^−2^), which is in agreement with the muographic results. In VSAB, on the contrary, the thickness of the tephra constantly increased during the current observation period. From the topographic changes, the estimated total mass (and mass per unit area) increased in VSAB during the period between October 2014 and October 2016 was 4.3 × 10^8^ kg (2.0 × 10^4^ kg m^−2^) (considering the upheaved area of 21,609 m^2^, and assuming a density of 2.0 g cm^−3^), which was also in agreement with the muographic results. Figure 7 shows the time-sequential images of the mass distribution plotted relative to the mass at the beginning of this observation campaign. In this figure in which a photograph of Sakurajma was overlaid, the volcanic column has risen from Showa Vent.

In conclusion, we developed a tephra deposition monitor and showed its potential by conducting the tephra fallout measurements with muography. By assuming the average density of the deposit was 2.0 g cm^−3^, the muographically estimated total mass (and mass per unit area) of the deposits accumulated between December 1, 2014 and November 20 were respectively 3.3 ± 1.4 × 10^8^ kg (1.2 ± 0.5 × 10^4^ kg m^−2^) and 3.9 ± 0.9 × 10^8^ kg (1.8 ± 0.4 × 10^4^ kg m^−2^) in VAB and VSAB. These values were compared with the values estimated from the airborne measurements and were in agreement with the airborne results within the statistical errors. Inversely, this agreement indicated that under this assumption for the average density of the deposit, the deposit thicknesses measured with muography were in agreement with the airborne results. The results showed that the current system is applicable to remotely monitoring the temporal growth of tephra deposits in the vicinity of the crater from a distance. By enlarging the size of the detector to collect a sufficiently large amount of muons in more limited timeframe, it would be possible to measure the amount of the volcanic deposit resulting from each targeted volcanic eruption, and to improve hazard mitigation and ultimately to estimate the potential volumes of future RTL.

## Methods

### Scintillation-based muography observation system (SMOS)

Detailed descriptions about the SMOS used for the current data collection are described elsewhere^38^, and thus only its basic concept will be briefly introduced here. The SMOS consisted of six layers of segmented scintillation detectors and five layers of lead-made-stainless steel covered radiation shields. Each scintillation detector consisted of 15 horizontally and 15 vertically aligned 1,500-mm long and 100-mm wide plastic scintillator strips, and thus the detector’s positioning resolution (Δ*X*, Δ*Y*) was ± 50 mm with a total active area of 2.25 m^2^. Each scintillator strip was connected to the photomultiplier tube (PMT) for signal reading. The distance between the furthest upstream and downstream detectors was 3,000 mm, and thus the angular resolution of the SMOS was ± 16.5 mrad and ± 33 mrad respectively for 75% and 100% of the incoming muons for both of the azimuth (*φ*) and elevation (*θ*) angles with a total viewing angle of − 460 mrad < *φ* < 460 mrad and 0 mrad < *θ* < 460 mrad. This angular resolution corresponded to a spatial resolution of ± 35 m (75%) and ± 69 m (100%) at the closest rim of Vent B, and ± 45 m (75%) and ± 89 m (100%) at Showa Vent. The lead-made radiation shields, which had a total thickness of 500 mm (100 mm × 5 layers) and were covered with stainless steel cases (20 mm × five layers, i.e., 100-mm thick in total) were used for the particle selection, which was sufficient to reject almost all hadronic and electromagnetic background particles that passed through the detector. Facing Sakurajima volcano, the SMOS measured the muons after passing through the volcano, while concurrently recording the unobstructed muons passing through open sky above the ridge of the mountain. The SMOS readout module^45^ processed signals from the detectors and generated the track information. The detector signals were received by the comparators and then were digitized after they were compared with the threshold voltage. An event filter played a role of a coincidence unit that selected events from the sampled signals and generated the information on each possible muon path. Signals from six layers of detectors that consisted of 180 scintillator strips in total (30 strips per one layer times 6 layers) were processed by a field programmable gate array (FPGA). These data were read by the network processor and accessed by a local data acquisition (DAQ) computer to download them. This local DAQ computer communicated with the database server at a remote place via an internet connection.

### Remote online-monitoring-system

The current real-time tephra monitoring system consisted of four main elements. These were (a) the SMOS located at the observation site including the readout module, (b) the DAQ computer, (c) the server to register, manage and visualize these data, and (d) a web browser installed on a remote computer. The component (c) was the core element of the current monitoring system. It was operated on a dedicated internet cloud service with the following specifications: (a) 6 cores of a central processing unit (CPU), (b) 20 gigabytes (GBs) of a memory, and (c) 800 GB of hard disk drive (HDD) space for processing and visualizing the SMOS data in real time. The operating system used was a 64-bit Canonical Ubuntu Linux 16.04LTS (Xenial Zerus). The database constructed in the server consisted of four kinds of data: (1) event data, (2) experimental condition data, (3) statistical information data, and (4) the Muogram Library. The event data were the compressed data generated by the SMOS readout module. The experimental condition data were configured to the actual setup of the SMOS (the distance between upstream and downstream detectors, number of layers used for analysis, and width of scintillator strips) at the observation site (SMO). Statistical information data that included hourly and daily event rate plots for each layer were automatically generated from the event data by incorporating the experimental condition data. These data could be used for monitoring the PMT’s condition of the detector. The event and experimental condition data were transferred to the ‘Muogram Generator’ to generate the track distribution on a φ-θ plane as a two-dimensional matrix that represents the number of muon tracks as a function of the elevation and azimuth angles) every 10 min automatically in the background. The generated track distribution was subsequently registered to the ‘Muogram Library’. These 10-min muograms could be aggregated later by choosing the specific period and angular region for an offline analysis. The time sequential plots of the muon count rate could be manually generated on this online monitoring system by setting the data aggregation period. In these plots, the volcanic column heights were automatically incorporated and plotted on these time sequential plots so that the comparison between the muon count rate and the volcanic activities could be easily compared. The information pertaining to the volcanic column height was automatically downloaded from the Japan Meteorological Agency (JMA) website^28^ and stored in the database. The rendering software visualized the muograms and time sequential plots enabled users to download the generated images.

### Muographically averaged densimetric/geometric thickness (MADT/MAGT)

By combining the cosmic muon’s energy spectrum^20^ and muon’s range-energy relationship^21^, the transmitted muon flux, *Φ* (*X*, *θ*, *ϕ*), can be derived. If the average density (*ρ*) of the target object is given, the densimetric rock thickness, *X*, can be defined by *ρx*, where *x* is the geometrical rock thickness. Once *X* is given, the minimum energy, *E*_c_, of muons that can escape from the target object can be calculated by using the range-energy relationship. *Φ* (*X*, *θ*, *ϕ*) can be then derived by integrating the muon’s energy spectrum *I*(*E*, *θ*) over the energy range between *E*_c_ and infinity.

3 $$\Phi \left( {\theta ,\phi } \right) = \mathop \smallint \limits_{{E_{c} \left( {\theta ,\phi } \right)}}^{\infty } I\left( {E, \theta } \right)dE$$

Since the observation apparatus has an angular resolution (*Δθ*, *Δϕ*), the transmitted muon flux measured in one image pixel is an integration of the flux of the muons that had passed through different regions in the target object. Therefore, the muon’s path length, *x*, in the target object varies as a function of arriving angles (*θ*, *ϕ*), and thus Eq. (3) must be calculated for different *θ* and *ϕ*, and averaged over the angle range between *θ* ± *Δθ* and *ϕ* ± *Δϕ* to derive the averaged transmitted flux, < *Φ* > , that can be directly compared with the observed flux in the image pixel. Inversely, if < *Φ* > is given, the muographically averaged densimetric thickness (MADT), < *X* > , can be uniquely determined by inserting < *Φ* > into Eq. (3) to derive averaged cutoff energy, < *E*_c_ > , that is directly compared with the energy-range relationship. Since the MADT is the column density averaged over the angle range (*θ* ± *Δθ* and *ϕ* ± *Δϕ*), if the distance between the detector and the target object (*D*) holds the following conditions:

4-1 $$D \gg D\Delta \theta ,$$

4-2 $$D \gg D\Delta \phi ,$$

4-3 $$D \gg L,$$

*D*^2^*ΔθΔϕ* < *X* > gives the total mass of the volume within the angle range, *θ* ± *Δθ* and *ϕ* ± *Δϕ*, where *L* is the typical thickness of the target object. The muographically averaged geometric thickness (MAGT) is defined by < *X* > / *ρ*. The MAGT is therefore different from the arithmetically averaged rock thickness.

### Atmospheric effects in the muographic measurements

(A) Rainfall effects. The annual rainfall in Kagoshima city, Japan is 2.3 × 10^3^ mm. The typical rainfall speed is 10 m/s. Therefore, the density increase in the atmosphere due to rainfalls, being averaged over 1 year is 7 × 10^−3^ g/m^3^, which is far below the detection limit (~ 10 kg/ m^3^) of the current observation. (B) Volcanic ash effects. The deposit area was at least 2 × 10^4^ m^2^; hence average tephra falls of 2.15 × 10^5^ kg/m^2^ in 720 days. Ash-fall speed is at an order of 10 m/s. Therefore, the density increase in the atmosphere due to ash falls, being averaged over 720 days is 0.3 g/m^3^ that is also far below the detection limit of the current observation. This estimate can be deviated by an order of magnitude, but it doesn’t affect the consequence of the current work.

### Normalization of the data

**Figure 8:**
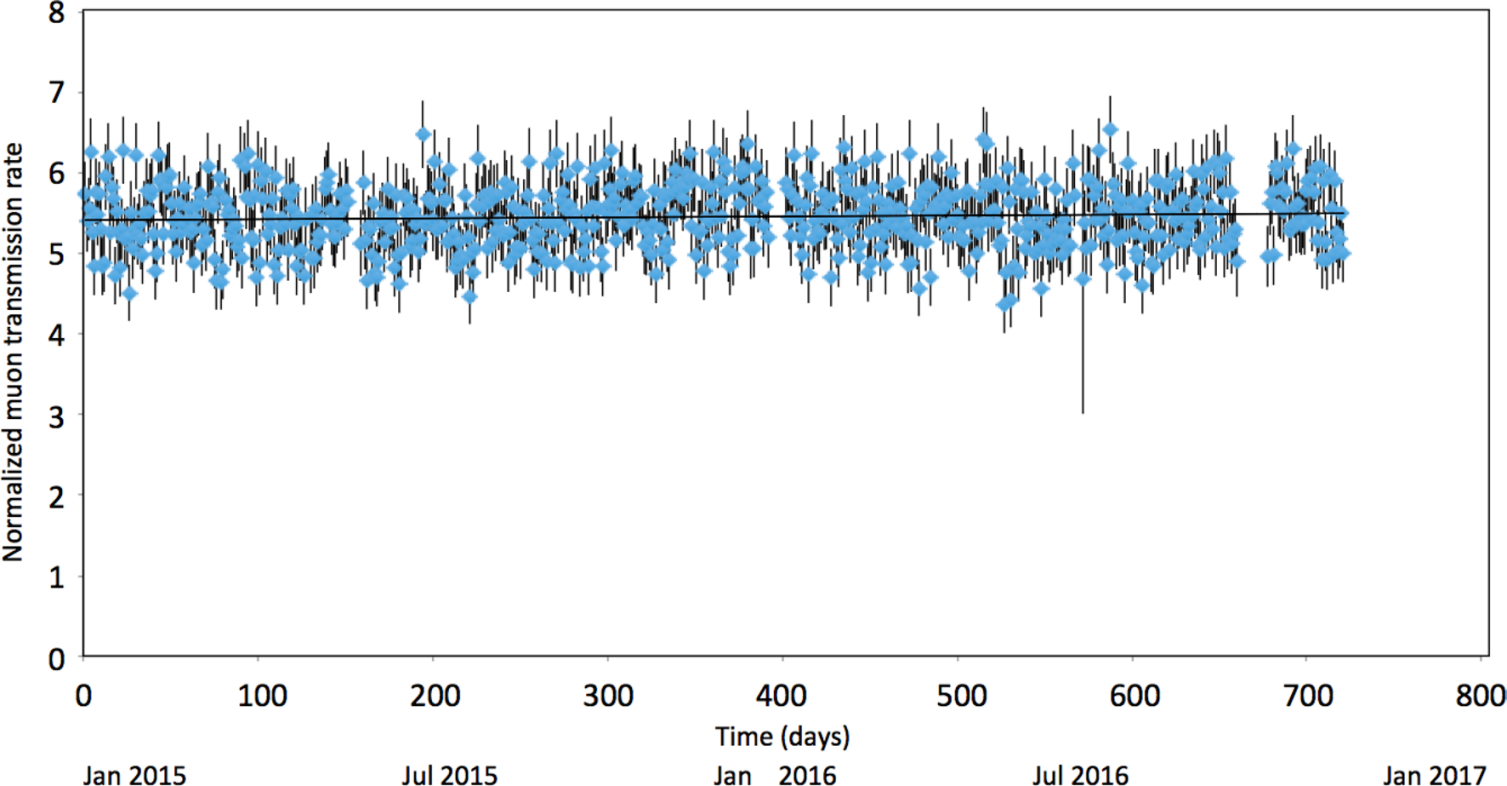
Normalization result for the muon data within the angular region that covers the entire mountain. The vertical bars associated with these data points are error bars (1 standard deviation). The horizontal line shows the maximum likelihood fitting result.

In this work, muons arriving from the direction above the ridge of the mountain (open-sky muons) were used for normalizing the count rate of the 5 angular regions (NM, VAB, VSAB, MAR, and sky). The time-sequential plots of muon counts collected from each angular region were respectively divided by the time-sequential plot of the open-sky muons and compared. The seasonal variations of the near-horizontal open-sky muon flux are generally small because the low-energy muons decay before arriving the sea level. However, the muon counts were slightly modulated due to the temporal variations of the detector condition including a gain drift of the photomultiplier tubes during a long period of operation. The current normalization technique cancels this effect. Figure 8 shows the normalization result for the muon data within the angular region that covers the entire mountain. The maximum likelihood fitting result (the horizontal line in Fig. 8) was *R* = 10^−4^ *t* + 5.4167, where *R* is the normalized muon ransmission rate and *t* is time in units of day, meaning that *R* at the beginning of the observation and *R* at the end of the observation varied by less than 0.2%. Also, no significant variations were found near the dormant region (NM) as can be seen in Fig. 4A.
